# How basic-level objects facilitate question-asking in a categorization task

**DOI:** 10.3389/fpsyg.2015.00918

**Published:** 2015-07-10

**Authors:** Azzurra Ruggeri, Markus A. Feufel

**Affiliations:** ^1^Center for Adaptive Behavior and Cognition, Max Planck Institute for Human DevelopmentBerlin, Germany; ^2^Department of Psychology, University of California, BerkeleyBerkeley, CA, USA; ^3^Department of Anesthesiology and Operative Intensive Care Medicine, Charité University Medicine BerlinBerlin, Germany

**Keywords:** binary categorization, information search, question-asking, inclusiveness, development

## Abstract

The ability to categorize information is essential to everyday tasks such as identifying the cause of an event given a set of likely explanations or pinpointing the correct from a set of possible diagnoses by sequentially probing questions. In three studies, we investigated how the level of inclusiveness at which objects are presented (basic-level vs. subordinate-level) influences children's (7- and 10-year-olds) and adults' performance in a sequential binary categorization task. Study 1 found a robust facilitating effect of basic-level objects on the ability to ask effective questions in a computerized version of the Twenty Questions game. Study 2 suggested that this facilitating effect might be due to the kinds of object-differentiating features participants *generate* when provided with basic-level as compared to subordinate-level objects. Study 3 ruled out the alternative hypothesis that basic-level objects facilitate the *selection* of the most efficient among a given set of features.

## Introduction

To be awarded the First Class Boy Scout badge in 1911, boys were required to identify “from observation six species of wild birds by their plumage, notes, tracks, or habits” (Boy Scouts of America, 1911). For preparation, the *Boy Scouts Handbook* instructed scouts to “notice the ‘range’ of birds in your reference book” and then look for “a match… by first examining the size of the bird (for example smaller than wren or larger than crow), then the location where the bird is observed (near ground or high up), then the color” (Boy Scouts of America, 1911). This is an example of a binary sequential categorization task, which can be solved by sequentially asking binary yes/no questions (e.g., Is the bird high up?; Is it smaller than wren?), to rule out non-target objects and converging on the target object in as few questions as possible. In this article, we present three studies in which we examine how 7- and 10-year-old children, as well as adults, seek information to solve a sequential binary categorization task. In particular, we investigate how the level of inclusiveness at which objects are presented influences participants' question-asking.

### The process of categorization and the level of inclusiveness

There is ample research on the development of object categorization during infancy and early childhood, when children continue to encounter new objects and situations (Bornstein, 1984; Rakison and Oakes, 2003). In the literature, two main views on categorization have emerged. The first view focuses on the *process* of categorization, and stresses the fact that objects can be categorized in different ways. The main finding is that people categorize the same entities differently depending on instructions, contexts, and task demands (Schyns and Rodet, 1997). Even young children change categorization strategies depending on their familiarity with the objects (Oakes et al., 1996), the distribution of exemplars (Bornstein et al., 1976; Hund and Plumert, 2005; Oakes and Ribar, 2005), or the properties of the category entities (whether they are prototype exemplars or not, whether the exemplars are presented singly or in pairs) in their task environment (Bauer et al., 1995; Oakes et al., 1997; Younger and Furrer, 2003; Mareschal and Tan, 2007).

The second view, which we focus on in this study, centers on the *structure* of the objects to be categorized, often referred to as the level of inclusiveness at which the objects are presented. The level of inclusiveness reflects the hierarchical taxonomy of the objects, consisting of nested sets into which objects are organized (Berlin et al., 1973, 1974; Atran, 1990). For example, the category “animals” includes, among others, dogs, fishes and birds. The category “dogs” in turn includes, among others, Retrievers, Dalmatians, and Collies. In this taxonomy, the category “animals” is at a higher level of inclusiveness than the category “dogs,” which, in turn, is at a higher level of inclusiveness than the category “Retrievers,” which in turn includes different subcategories, such as Golden Retrievers or English Setters. In infant, child, and adult cognitive studies (e.g., Rosch, 1978; Mervis and Rosch, 1981; Herwig, 1982; Liu et al., 2001; Murphy, 2002; Ellis and Oakes, 2006) the different levels of inclusiveness have been referred to as *superordinate* (e.g., “animals”), *basic* (e.g., “dogs”), and *subordinate* level (e.g., “Retriever”). In this study we will be following this definition, although we recognize that the level of inclusiveness of a category cannot be defined in absolute terms, and that it depends, for example, on a person's level of expertise and cultural background (Dougherty, 1978; Tanaka and Taylor, 1991; Bornstein and Arterberry, 2010).

Many studies have supported an advantage of the basic level of inclusiveness for categorization in young children (Mervis and Crisafi, 1982; Mandler and Bauer, 1988; Bauer et al., 1995). According to ethnobiologists, this level occupies a psychologically privileged taxonomic position because it captures categories widely found in nature and affords the requirements for categorization, reasoning, and use within a biological system (Bulmer, 1974; Hunn, 1982; Ellen, 1993). Research in cognitive psychology has similarly shown that objects presented at the basic level are the first named and understood by children because they mirror natural kinds and the organization of our knowledge (Rosch, 1978; Mervis, 1987) and emphasize information about the basic structures, functions, and perceptual characteristics that characterize individual objects (Wisniewski and Murphy, 1989). Also, “research in both language acquisition and categorization converges on the precedence of basic-level words and basic-level categories … children can categorize and label objects at the basic level … long before they can do so at other levels” (Liu et al., 2001). For example, Rosch and colleagues found that preschoolers matched adults in classifying objects into basic-level categories such as shoes, chairs, and cars but not into superordinate categories such as clothes, furniture, or vehicles (Rosch et al., 1976).

### The twenty questions game and the present studies

Previous research investigating children's information search has used versions of the Twenty Questions game. In its experimental version, participants are presented with a fixed number of alternatives and their task is to identify the target alternative by asking as few questions as possible. Only yes/no questions are allowed. This paradigm has been used to study children's question-asking strategies both in causal learning tasks (Ruggeri and Lombrozo, 2014; submitted), where participants have to identify the cause of an observed event, and in sequential binary categorization tasks, either generating the questions from scratch (Mosher and Hornsby, 1966; Denney and Denney, 1973; Herwig, 1982; Chouinard, 2007; Legare et al., 2013; Ruggeri et al., 2015) or selecting them from a list of provided alternatives (Nelson et al., 2014).

Overall, experimental results show that the ability to ask effective questions undergoes a large developmental change from age 4 to 10. Specifically, younger children tend to ask questions concerning particular objects (i.e., so-called *hypothesis-scanning* questions, such as “Is it a dog?”), whereas older children and adults tend to ask questions about object features that help rule out more than one object at a time (i.e., so-called *constraint-seeking* questions, such as “Does the animal fly?”). The observed transitions are explained as a shift from a perceptual focus on individual stimuli and objects to a tendency to recognize object-general features that can be used to group and cluster similar objects into categories (e.g., flying animals vs. nonflying animals) and, by this, to guide categorization strategies (Mosher and Hornsby, 1966).

Previous work have demonstrated that the Twenty Questions game can provide a rich source of data that reflects developmental changes in children's strategies for inquiry. Moreover, although the Twenty Questions game may appear quite artificial, the problem of sequential binary information search is a general one, encountered throughout the lifespan. For instance, a similar process can be used for medical diagnoses: In emergency medicine, resident physicians learn to check for the presence or absence of certain physiological changes to rule out lethal conditions that can be associated with a particular complaint (e.g., Green and Mehr, 1997; Hamilton et al., 2003). Also, various real world decision-making, categorization, and causal inference tasks have been modeled with fast and frugal trees that involve sequential, binary branching (see Berretty et al., 1999; Martignon et al., 2008). Thus, studying children's performance on a 20-questions task is a good compromise between experimental tractability and real-world generalizability.

Taking Rosch et al.'s (1976) results into account, Herwig (1982) tested preschoolers, first, second, and fifth graders on the Twenty Questions game. Herwig hypothesized that performance would be best when children are given objects represented at the subordinate level (e.g., sportscar, van, raincoat, jacket) because they are familiar with features that differentiate the basic-level categories to which those objects belong (e.g., cars and coats). These higher order features are necessary to ask effective questions in the Twenty Questions game. When given objects represented at the basic level (e.g., car and coat), they should perform worse because children are less familiar with features that differentiate between superordinate categories to which those objects belong (e.g., vehicles and clothes). Indeed, children's performance in the Twenty Questions game improved when given subordinate-level objects, but only if they had the chance to group the objects into categories before starting the game. Without training, in contrast, children's performance was best when objects were represented at the basic level. This suggests that basic-level objects help children generate higher order features that lead them to ask effective questions in the Twenty Questions game, without requiring prior training.

In this paper, we sought to replicate, extend and explain the facilitating effect of basic-level objects on categorization performance in a sequential binary categorization task. In particular, we investigated the effect of the level of inclusiveness (basic-level vs. subordinate-level) on the ability to ask effective questions in a computerized version of the Twenty Questions game (Study 1). We further attempted to disentangle the effect of the level of inclusiveness on two crucial abilities necessary to ask effective questions: In Study 2, we investigate participants' abilities to *generate* features useful for categorization, that is, features ruling out classes of objects (e.g., Does it have four legs?) rather than individual objects (e.g., Is it a dog?). In Study 3, we investigated participants' abilities to *select* the most efficient among a given set of questions.

To assess the generality of our findings, we tested children with different stimuli in two domains (animals and professions) and elaborated the developmental trend in how 7-year-olds generate, select and use object-differentiating features as compared to 10-year-olds and adults. These age ranges were motivated by prior research suggesting a developmental shift in children's strategies for inquiry between the ages of 7 and 10 (Mosher and Hornsby, 1966; Ruggeri and Lombrozo, 2014; submitted).

## Study 1

Study 1 investigates developmental trends in inquiry strategies by manipulating the level of inclusiveness used to represent objects in a computerized version of the Twenty Questions game. We extended earlier findings by comparing the performance of 7- and 10-year-olds with adults. Moreover, to assess the generality of our findings, we tested children with different stimuli in two domains—animals and professions.

### Hypotheses

#### Main effect of level of inclusiveness

Based on prior research (Herwig, 1982), we expected that, in absence of training, objects presented at the basic level of inclusiveness would trigger questions with higher informational value for categorization.

#### Main effect of domain

We chose the animals and the professions domains because children and adults likely differ in how familiar they are with animals and professions. Children, in contrast to adults, naturally focus on perceptual features of objects (Wartella, 1979; Flavell, 1985; John and Sujan, 1990; Springer, 2001), and animals' morphological features are usually learned in school and often sufficient to differentiate between them. Differences among professions, on the other hand, are less perceptual in nature and are mainly learned later in life. Thus, with respect to object domains, we expected a decline in performance for children asked to categorize professions, but not for adults.

#### Main effect of age group

With respect to age differences we expected, based on previous research (Mosher and Hornsby, 1966; Ruggeri and Lombrozo, submitted), that adults would perform better than older children, and older children better than younger children, in terms of number, type, and informational value of the questions asked.

### Method

#### Participants

We tested 53 7-year-old children (15 females, *M*_age_ = 7.1 years; *SD* = 0.8), 61 10-year-old children (30 females, *M*_age_ = 9.5 years; *SD* = 0.5), and 94 adults (36 females, *M*_age_ = 27.9 years; *SD* = 1.8). Two additional 7-year-old children were excluded from the analyses because they did not know one or more of the objects used in the study. All children were recruited from the Istituto Sacro Cuore primary school in Livorno, Italy. Adult participants were recruited from the University of Pisa, Italy. The experimental procedures were approved by the ethics committee of the Max Planck Institute for Human Development, and all the parents of the children involved, as well as the teachers and the schools' Institutional Review Board, were informed and consented (in written form) to let the children participate prior to data collection. Children participants were asked to give their assent to participate. Adult participants also consented to participate in written form. All participants were free to withdraw from the experiment at any time.

#### Materials

For Study 1, we used word labels of 20 objects from two domains—animals and professions—at both the basic and subordinate level (i.e., 40 objects per domain). We decided against using pictures or icons as they may trigger (perceptual) features that participants were not thinking of themselves.

To ascertain that children were familiar with the word labels (see Appendix 1 in Supplementary Material for the Italian originals and their English translation), we showed the labels in random order to an independent sample of 20 6- to 8-year-old children and 20 adults. During this pilot test, we asked children and adults to indicate if they had heard of the animal/profession before, and to provide a short description. For the word labels at the subordinate level, children were also asked to name the basic level category to which they belong (e.g., “What kind of animal is a Dalmatian?” or “Which type of profession does a Dentist belong to?”). All children and adults had heard of all the word labels before and could provide a short description. For each of the word labels at the subordinate level, at least 64% (on average 74%) of the children and 95% of the adults (on average 98%) could correctly name the correspondent basic-level category.

#### Design and procedure

Participants were presented with 20 cards displayed on a computer screen, each presenting a label. Participants were randomly assigned to one of two experimental conditions based on the level of inclusiveness at which the objects were presented: basic-level condition (e.g., “dog” or “musician”) or subordinate-level condition (e.g., “Dalmatian” or “flute player”). All objects presented to participants are listed in Appendix 1 (Supplementary Material; Italian originals and English translation), sorted by condition and domain. Domain was a between-subjects factor; that is, the objects were taken from either the animal or the profession domain. Given that younger children took between 30 and 60 min to complete the task, presenting both domains would have taken too much time for them to keep up concentration.

Before starting the experimental session, the experimenter read aloud all the objects on the screen, making sure participants were familiar with all of them (exclusion criterion). The computer randomly selected one object from the set of 20 as target object, and was displayed on the experimenter's but not the participants' screen. Participants were instructed to ask the experimenter yes/no questions to identify this object. They were told that open questions such as “What kind of food does the animal eat?” were not allowed, and therefore would not be answered. Participants' questions were typed on the computer by the experimenter and became visible on the right side of the participants' screen, where they remained visible until the end of the round. With this memory aid, participants did not have to keep track of what they had asked previously. After answering a yes/no question, the experimenter crossed out the objects the answer ruled out by clicking on the corresponding buttons on the experimenter's screen. The eliminated objects turned darker on both screens to help participants focus on the remaining objects. A round was over when only one object was left or the target object was identified.

To emphasize the game character and motivate participation, participants were given 60 points at the outset and had to pay 1 point for each question they asked. Participants were given 5 points for identifying the target object. The score was continually updated and appeared in the upper right corner of the screen. Participants were told that the three players with the highest score would be awarded a box of colored pencils (children) or a 20-Euro Amazon gift card (adults).

#### Dependent measures

Results were analyzed with respect to developmental differences on three outcomes: (1) the number of questions needed to reach the solution; (2) the questions' effectiveness, measured in terms of information gain; and (3) the type of questions. We will explain Outcomes 2 and 3 in turn.

Following previous research on the Twenty Questions game (Nelson et al., 2014; Ruggeri et al., 2015), we used information gain to measure question effectiveness (for other examples in psychology see (Oaksford and Chater, 1994, 1996; Nelson, 2005). As defined within the framework of information theory, information gain (Lindley, 1956) refers to the expected reduction of entropy (Shannon, 1948), that is, to the expected reduction of uncertainty obtained by a particular question. Intuitively, a good question is able to reduce the uncertainty concerning the target object by narrowing down the number of alternatives left. Imagine 15 animals and the question “Can this animal fly?” that splits the 15 objects into 5 flying and 10 nonflying animals. To measure its information gain *I*, the posterior entropy is subtracted from the prior entropy. The prior entropy for 15 objects *H*_prior_ = log_2_(15) = 3.91. If the target animal is a bird, then the posterior entropy *H*_fly_ = log_2_(5) = 2.32; if not, *H*_not fly_ = log_2_(10) = 3.32. Thus, on average the posterior entropy *H*_posterior_ = (5/15 ^*^ 2.32) + (10/15 = 3.32) = 2.98, and the question's information gain *I* = *H*_prior_ − *H*_posterior_ = 3.91 − 2.98 = 0.93. According to this measure, the maximum information gain is 1.0, obtained by a question that splits the remaining objects in half.

Regarding Outcome 3, we followed the previous literature (Mosher and Hornsby, 1966; Denney and Denney, 1973; Herwig, 1982) and coded the questions as hypothesis scanning, constraint seeking, or pseudoconstraint seeking. Hypothesis-scanning questions target particular objects, as in “Is it the dog?,” and are able to eliminate, if wrong, only one object among the remaining ones. Constraint-seeking questions target object-general features, as in “Does it have four legs?,” and are able to rule out groups of objects. Pseudoconstraint-seeking questions target object-specific features and rule out only one object at a time, as in “Does it bark?”

The three types of outcomes are interdependent. In particular, both hypothesis-scanning and pseudoconstraint-seeking questions usually yield lower information gain than constraint-seeking questions so that, on average, inquiry strategies based on the former would require more questions to reach the solution. In fact, hypothesis-scanning and pseudoconstraint-seeking questions are a degenerate case of constraint-seeking questions, in which the space of possible solutions is partitioned into two sets: One set containing a single hypothesis and the other containing everything else. However, the relative advantage of a constraint-seeking approach is not fixed, and in some cases the informativeness of a hypothesis-scanning question can be equal or even higher than constraint-seeking alternatives. For example, presented with only two equally likely candidate hypotheses, constraint-seeking questions will be no more informative than hypothesis-scanning questions. Moreover, when members within the set of candidate solutions are not all equally likely, a hypothesis-scanning question that targets a single very likely hypothesis (e.g., one that has a 50% probability of being true) can be more informative than a constraint-seeking question that differentiates an even number of hypotheses, but where the summed probability of those in one partition is small (Ruggeri and Lombrozo, submitted).

We considered the three outcome variables as complementary measures of the quality of inquiry strategies, combining both quantitative and qualitative accounts of participants' categorization performance. Such a comprehensive analysis of categorization performance has, to our knowledge, not yet been conducted.

### Results

For each dependent measure, we ran a univariate analysis of variance (ANOVA), with age group, inclusiveness, and object domain as between-subjects variables. All main effects and interactions were tested, but we report only significant effects.

#### Number of questions

The minimum number of questions necessary to reach the solution in this task is 4, assuming that participants can generate, at each step of the inquiry process, a question splitting the remaining set of objects in two equally large sets. The maximum number of questions one might need to reach the solution is 20, assuming a participant asking exclusively hyothesis-scanning questions who identifies the solution at the very end of the search.

**Figure 1:**
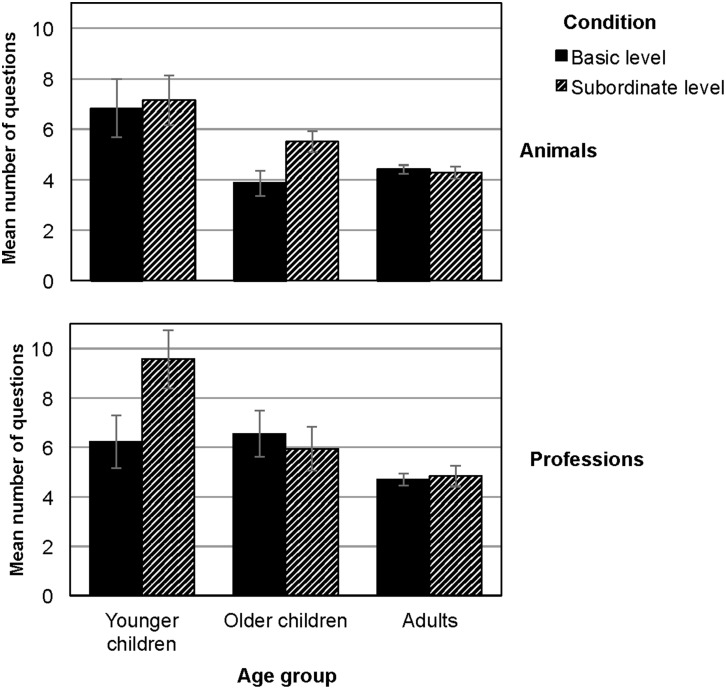
**Number of questions needed to reach the solution, displayed by domain (animals, professions) and condition (basic level, subordinate level)**. Error bars indicate one SEM in each direction.

Inclusiveness did not affect the number of questions asked. Although participants needed fewer questions to reach the solution when objects were presented at the basic level (*M*_basic−level_ = 5.4; *SD* = 0.3) compared to the subordinate-level condition (*M*_subordinate−level_ = 6.2; *SD* = 0.3), the effect of level of inclusiveness was only marginally significant, *F*_(1, 207)_ = 2.95, *p* = 0.08, η^2^ = 0.02. As hypothesized, we found a main effect for age group, *F*_(2, 207)_ = 13.79, *p* < 0.001, η^2^ = 0.13. Bonferroni *post-hoc* tests revealed that the average number of questions asked by adults and older children did not differ (*M*_adults_ = 4.7; *SD* = 1.5; *M*_older_children_ = 5.7; *SD* = 2.9; *p* = 0.15), but both older children and adults asked fewer questions than younger children (*M*_younger_children_ = 7.8; *SD* = 4.6; *p* < 0.001; see Figure 1). We also found the hypothesized main effect for object domain, *F*_(1, 207)_ = 5.82, *p* = 0.03, η^2^ = 0.02. Participants asked more questions when they had to categorize professions (*M*_professions_ = 6.1; *SD* = 3.6) than animals (*M*_animals_ = 5.3; *SD* = 2.4).

#### Information gain

**Figure 2:**
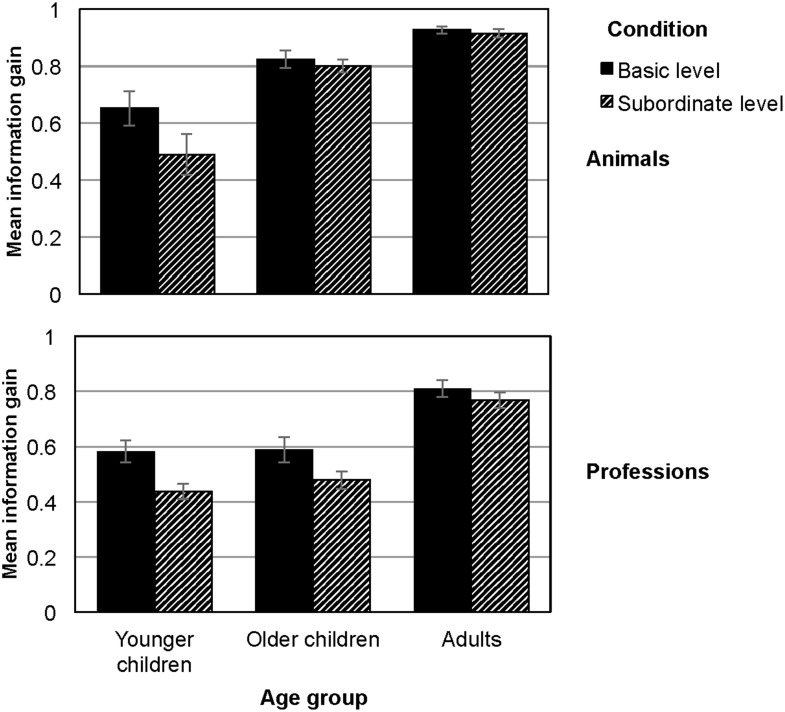
**Average effectiveness of participants' questions in terms of information gain, displayed by domain (animals, professions) and condition (basic level, subordinate level)**. Error bars indicate one SEM in each direction.

The analysis revealed the hypothesized main effect of inclusiveness, *F*_(1, 207)_ = 19.8, *p* < 0.001, η^2^ = 0.09. Participants asked questions with higher information gain when objects were presented at the basic level (*M*_basic−level_ = 0.8; *SD* = 0.2) compared to the subordinate-level condition (*M*_subordinate−level_ = 0.7; *SD* = 0.2, Figure 2). We also found the hypothesized main effect of object domain, *F*_(1, 207)_ = 56.4, *p* < 0.001, η^2^ = 0.22, with participants asking questions with higher information gain in the animal than in the profession domain (*M*_animals_ = 0.8; *SD* = 0.2; *M*_professions_ = 0.7; *SD* = 0.2). As for the number of questions, we found the hypothesized main effect for age group, *F*_(2, 207)_ = 95.7, *p* < 0.001, η^2^ = 0.49, with adults asking questions with higher information gain (*M*_adults_ = 0.9; *SD* = 0.1) than older children (*M*_older_children_ = 0.7; *SD* = 0.2, *p* < 0.001) and older children asking questions with higher information gain than younger children (*M*_younger_children_ = 0.5; *SD* = 0.2, *p* < 0.001).

Moreover, we found an Age × Domain interaction, *F*_(2, 207)_ = 9.9, *p* < 0.001, η^2^ = 0.09. The difference in information gain between the animal and profession domain was stronger for adults [animals: *M*_adults_ = 0.9; *SD* = 0.1; professions: *M*_adults_ = 0.8; *SD* = 0.1; *t*_(92)_ = 4.2, *p* < 0.001] and older children [animals: *M*_older_children_ = 0.8; *SD* = 0.1; professions: *M*_older_children_ = 0.5; *SD* = 0.2; *t*_(59)_ = 8.2, *p* < 0.001] than for younger children [animals: *M*_younger_children_ = 0.6; *SD* = 0.2; professions: *M*_younger_children_ = 0.5; *SD* = 0.2; *t*_(51)_ = 1.9, *p* = 0.085].

#### Question type

**Figure 3:**
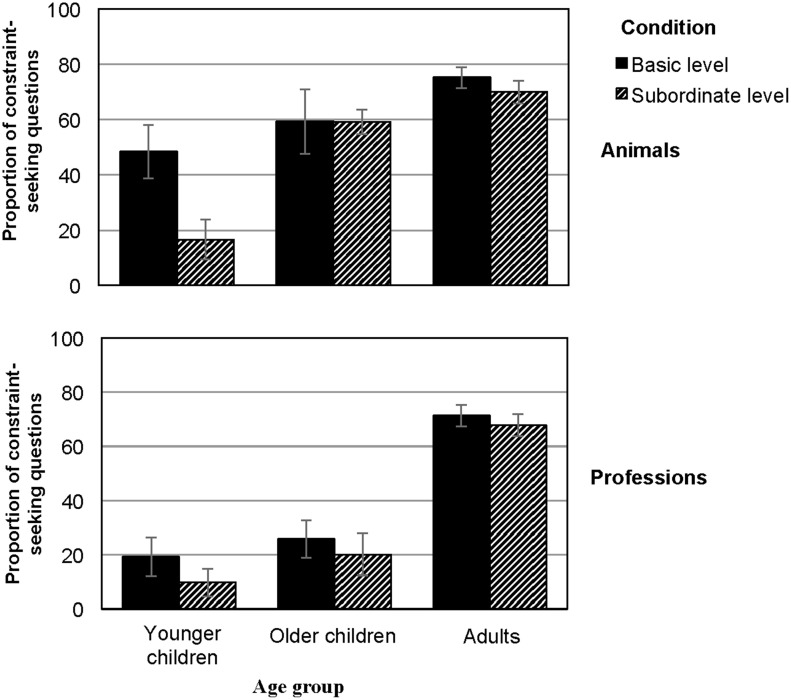
**Percentage of participants' constraint-seeking questions, displayed by domain (animals, professions) and condition (basic level, subordinate level)**.

Because we were primarily interested in the influence of object inclusiveness on participants' abilities to ask constraint-seeking questions (i.e., questions that ask about object-general features and therefore rule out more than one object at time), we report only the analysis of the proportion of constraint-seeking questions relative to the total number of questions for the sake of brevity (see Figure 3).

As hypothesized, we found main effects for all between-subjects variables, that is, inclusiveness, *F*_(1, 207)_ = 7.1, *p* = 0.008, η^2^ = 0.04, object domain, *F*_(1, 207)_ = 24.9, *p* < 0.001, η^2^ = 0.11, and age group, *F*_(2, 207)_ = 64.03, *p* < 0.001, η ^2^ = 0.40, on the proportion of constraint-seeking questions participants asked. The main effect of object domain showed that participants more readily generated constraint-seeking questions when asked to categorize animals (59%; *SD* = 27, Figure 3, top) than professions (45%; *SD* = 36, Figure 3, bottom). Bonferroni *post-hoc* analyses of the main effect of age group confirmed earlier research in that adults asked a higher proportion of constraint-seeking questions (72%; *SD* = 20) than older children (41%; *SD* = 32; *p* = 0.001), who asked a higher proportion of constraint-seeking questions than younger children (22%; *SD* = 30; *p* < 0.001). The main effect for inclusiveness showed that participants asked a higher proportion of constraint-seeking questions in the basic-level condition (58%; *SD* = 32) than in the subordinate-level condition (43%; *SD* = 34).

### Discussion of study 1

As hypothesized and consistent with previous incidental findings (e.g., Herwig, 1982), objects presented at the basic level facilitated the generation of effective questions in a binary sequential categorization task. Although the inclusiveness of objects only marginally impacted the number of questions asked (Ruggeri and Lombrozo, submitted), all age groups asked proportionally more constraint-seeking questions with higher information gain when objects were presented at the basic level. Conversely, when asked to categorize subordinate-level objects, participants asked fewer constraint-seeking questions with lower information gain, even though they could have technically asked the same constraint-seeking questions their peers had asked in the basic-level condition.

Object domains impacted categorization performance in that professions were more difficult to categorize than animals. Specifically, participants across all age groups needed more questions, and asked proportionally fewer constraint-seeking questions, when asked to categorize professions. The finding that older children and adults benefitted from basic-level objects more in the animals than in the professions domain indicates that the benefit of basic-level objects is contingent on domain knowledge.

In general, we confirmed our developmental hypothesis by showing that adults performed better than older children, and older children better than younger children.

In summary, results of Study 1 replicated previous studies by showing that objects presented at the basic level facilitate the generation of effective categorization questions across age groups. Study 2 and 3 are designed to deconstruct the facilitating effect of level of inclusiveness on participants' performances in the Twenty Questions task, by disentangling the effect of the level of inclusiveness on two crucial abilities necessary to ask effective questions: In Study 2, we investigated participants' abilities to *generate* features useful for categorization, that is, features ruling out classes of objects (e.g., Does it have four legs?) rather than individual objects (e.g., Is it a dog?). In Study 3, we investigated participants' abilities to *select* the most efficient among a given set of questions.

## Study 2

In Study 2 we examined the amount and kinds of object-differentiating features the different levels of inclusiveness trigger in younger children, older children, and adults. We asked participants to generate features that would help them differentiate a given object (e.g., a dog) from other objects within the same superordinate category (i.e., animals). A similar methodology has been used to address the effects of expert knowledge on the basic level categorization (Palmer et al., 1989; Tanaka and Taylor, 1991; Johnson and Mervis, 1997; Medin et al., 1997). We examined the types of differentiating features that are triggered when objects are presented at the basic level (e.g., dog) or the subordinate level (e.g., Dalmatian).

### Hypotheses

#### Main effect of level of inclusiveness

Good performance in sequential binary categorization tasks requires features that help rule out more than one object at a time. These features must be object general and describe properties shared by more than one object (e.g., it is a mammal) rather than being specific to only one object (e.g., it barks). Objects presented at the subordinate level (e.g., Dalmatian) are taxonomically less general than basic-level objects (e.g., dog) and imply additional object-specific features. We therefore hypothesized that participants in the subordinate-level condition generate more object-specific features (e.g., “Dalmatians have black and white spots” rather than “Dalmatians have four legs”), whereas in the basic-level condition they tend to generate more object-general features (e.g., “Dogs have four legs”).

#### Main effect of domain

As in Study 1, to assess the generality of our findings, we tested children with stimuli from two domains—dog from the animals domain and doctor from the professions domain. Consistent with the results from Study 1, we expected that participants would generate more differentiating features in the animals compared to the professions domain.

#### Main effect of age group

Assuming developmental differences in knowledge about differentiating features, we expected, as in Study 1, that adults generate more features than older children, and older children more than younger children.

### Method

#### Participants

We tested 43 7-year-old children (21 females, *M*_age_ = 7.5 years; *SD* = 0.5), 60 10-year-old children (23 females, *M*_age_ = 9.8 years; *SD* = 0.6), and 33 adults (15 females, *M*_age_ = 25.5 years; *SD* = 5.4). All children were recruited from the Fondazione San Carlo Borromeo primary school in Livorno, Italy. Adult participants were recruited from the University of Pisa, Italy.

#### Design and procedure

Participants were given one object from each domain in random order and were randomly assigned to one of the two experimental conditions based on inclusiveness: the basic level condition (participants received “dog” and “doctor”) or the subordinate level condition (participants received “Dalmatian” and “dentist”). Participants in both conditions were instructed to “name all the features that make this animal [profession] different from the following other animals [professions]” (see Appendix 2 in Supplementary Material for the original instructions in Italian) and were given the list of the other 19 animals/professions as considered in Study 1, presented at the same level of inclusiveness. For each object, participants had 5 min to list features. Then, children received a box of colored pencils, and adult participants were entered in a lottery for a chance to win a 30-Euro Amazon gift card.

#### Dependent measures

We analyzed the features participants generated with respect to (a) the *number* of features and (b) the *type* of features, with types being object-general and object-specific. Object-general features describe properties of the given animal/profession shared by some of the other animals/professions listed (e.g., “dogs/Dalmatians have four legs”; “doctors/dentists have a university degree”). Object-specific features are specific to the given animal/profession (e.g., “dogs bark” or “Dalmatians have black and white spots”; “doctors operate” or “dentists fix teeth”). When coding the data, we identified a third type of feature, which was used by younger and a few older children. Such features did not differenciate the given from other objects but simply stated what a given object was not (e.g., “It is not a snake” for the dog, or “It is not an architect” for the doctor). We refer to this type of feature as a *contrasting* feature. The generated features were coded by the experimenter, a student assistant blind to the experimental hypotheses, immediately after the session. All questions were additionally and independently coded by a second student assistant, blind to the experimental hypotheses, resulting in total agreement of Kappa = 0.849, *p* < 0.001. In the few cases where the two raters did not agree, a third rater was consulted to achieve consensus.

### Results

#### Number of features

We ran a repeated-measures analysis of variance (ANOVA) with domain as within-subject factor and age group and level of inclusiveness as between-subjects variables. We found a main effect of inclusiveness, *F*_(1, 97)_ = 5.4, *p* = 0.022, η^2^ = 0.05, with participants generating more features when given basic-level objects (*M*_basic−level_ = 3.9; *SD* = 1.4) than subordinate-level objects (*M*_subordinate−level_ = 3.3; *SD* = 1.3), a main effect of domain, *F*_(1, 97)_ = 37.4, *p* < 0.001, η^2^ = 0.28, with participants generating more features when asked to differentiate dogs from other animals (*M*_animals_ = 4.1; *SD* = 1.7) than doctors from other professions (*M*_professions_ = 3.1; *SD* = 1.6). A significant interaction of Domain × Inclusiveness, *F*_(1, 97)_ = 9.9, *p* = 0.002, η^2^ = 0.1, showed that participants identified more features in the basic-level than in the subordinate-level condition only in the animal domain [*M*_basic−level_ = 4.7; *SD* = 1.7; *M*_subordinate−level_ = 3.5; *SD* = 1.4; *t*_(101)_ = 3.8, *p* < 0.001], but not in the profession domain (*M*_basic−level_ = 3.2; *SD* = 1.4; *M*_subordinate−level_ = 3.0; *SD* = 1.6; *p* = 0.624).

We also found a main effect of age group, *F*_(2, 97)_ = 4.7, *p* = 0.011, η^2^ = 0.09. Bonferroni *post-hoc* tests showed that adults and older children generated about the same number of features (*M*_adults_ = 3.9; *SD* = 1.4; *M*_older_children_ = 3.9; *SD* = 1.6; *p* = 0.98), and both generated more features than younger children (*M*_younger_children_ = 3.0; *SD* = 0.7; *p* < 0.02).

#### Type of feature

**Figure 4:**
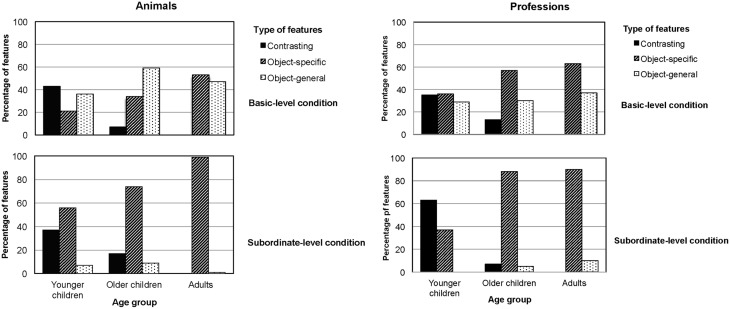
**Percentage of features (rounded) generated, displayed by age group, feature type (object-general, object-specific, and contrasting), and inclusiveness (basic level and subordinate level) in the animal domain (left panel) and the profession domain (right panel)**.

Figure 4 displays the proportions of the three types of features generated—object-general, object-specific, and contrasting features—based on domain and inclusiveness. We ran three repeated-measures ANOVAs with domain as the within-subject factor and age group and inclusiveness as between-subjects variables, considering in turn the proportions of object-general, object-specific, and contrasting features in relation to the total number of features participants had generated.

With respect to object-general features, we found a main effect of level of inclusiveness. Participants generated a higher proportion of object-general features in the basic-level condition (40%; *SD* = 25) than in the subordinate condition (5%; *SD* = 9), *F*_(1, 97)_ = 79.0, *p* < 0.001, η^2^ = 0.45. We also found a main effect of domain, with participants generating a higher proportion of object-general features in the animals domain (27%; *SD* = 33) than in the professions domain (18%; *SD* = 20), *F*_(1, 97)_ = 5.14, *p* = 0.026, η^2^ = 0.05. There was no main effect of age group on the proportion of object-general features (*p* = 0.227).

Correspondingly, we found a main effect of inclusiveness with respect to object-specific features. Participants generated a lower proportion of object-specific features in the basic-level condition (45%; *SD* = 30) than in the subordinate condition (75%; *SD* = 27), *F*_(1, 97)_ = 36.8, *p* < 0.001, η^2^ = 0.28. There was no effect of domain, but there was a main effect of age group on the proportion of object-specific features, *F*_(1, 97)_ = 19.19, *p* < 0.001, η^2^ = 0.29. Bonferroni *post-hoc* analyses revealed that adults and older children generated about the same proportion of object-specific features (*M*_adults_ = 76%; *SD* = 28; *M*_older_children_ = 63%; *SD* = 30; *p* = 0.082), and both generated more object-specific features than younger children (*M*_younger_children_ = 38%; *SD* = 28; *p*s < 0.001).

With respect to contrasting features (e.g., “It's not a snake”), we found a main effect of age group only, *F*_ (1, 97)_ = 3.33, *p* < 0.001, η^2^ = 0.39. Bonferroni *post-hoc* analyses revealed that adults and older children generated about the same proportion of contrasting features (*M*_adults_ = 0%; *SD* = 0; *M*_older_children_ = 11%; *SD* = 25; *p* = 0.102), and they both generated fewer contrasting features than younger children (*M*_younger_children_ = 45%; *SD* = 31; *p* < 0.001).

### Discussion of study 2

Asking participants to identify object-differentiating features, we found as hypothesized that, across all age groups, basic-level objects triggered proportionally more object-general features, which, in categorization tasks, help to rule out more than one object at a time and facilitate efficient identification of a target object. Although older children and adults generated more features than younger children, the proportion of object-general features did not differ across age groups but domains. Thus, whereas the effect of the basic level of inclusiveness is dependent on domain-specific knowledge, all age groups are similarly impacted by it.

To differentiate Dalmatian/Dentist from other animals/professions, participants could have named the same features as for the corresponding basic-level objects dog/doctor. However, when objects were presented at the subordinate level (e.g., Dalmatian), participants of all ages generated proportionally more object-specific features (“Dalmatians have black and white spots”), which can be used to rule out only one or a few objects at a time in categorization tasks. Thus, Study 2 suggests that categorization performance is in part facilitated or hindered by the objects' level of inclusiveness because it tends to trigger knowledge related to object-general or object-specific features, respectively.

From a developmental perspective, Study 2 showed that older children resembled adults in that both age groups were able to generate a similar number of object-differentiating features, which was larger than what younger children were able to generate. Moreover, older children and adults proportionally generated more object-specific features and fewer contrasting features than younger children. This seems to suggest that younger children lag behind their older counterparts in their ability to generate object-differentiating features, whereas older children more closely match adults' performance.

To summarize, the facilitating effect of the basic-level of inclusiveness on participants' performance in Study 1 may be rooted in the kind of features triggered by the different object levels. However, performance in a sequential binary categorization task depends not only on knowledge of differentiating features but also on the ability to identify the most effective among them. With Study 3 we investigate whether the level of inclusiveness also influences the ability to identify and select efficient questions.

## Study 3

### Hypotheses

If the effect of object inclusiveness does not help participants identify effective inquiry strategies, eliminating differences in knowledge of object-general features should make the effects of object domain and inclusiveness disappear, but not the developmental differences in categorization performance we observed in Study 1 (see also Mosher and Hornsby, 1966). To eliminate knowledge differences, we used the same setup as in Study 1, but instead of letting participants generate questions we provided a choice set made up of constraint-seeking, hypothesis-scanning, and pseudoconstraint-seeking questions, each with predefined effectiveness (i.e., information gain). Thus, participants did not need to generate features but only had to select the questions with the features they considered most effective.

### Method

#### Participants

We tested 30 seven-year-old children (13 females, *M*_age_ = 7.7 years; *SD* = 0.5), 30 ten-year-old children (14 females, *M*_age_ = 9.8 years; *SD* = 0.6), and 28 adults (12 females, *M*_age_ = 27.5 years; *SD* = 0.2). All children were recruited from the Fondazione Sacro Cuore primary school in Livorno, Italy. Adult participants were recruited from the University of Pisa, Italy.

#### Design and procedure

Participants were presented with a computerized version of the Twenty Question game similar to the one used in Study 1 and randomly assigned to one of two conditions: the basic-level or subordinate-level condition. They played the same experimental condition twice, once with objects from the animal domain and once with objects from the profession domain (see Appendix 1 in Supplementary Material). The order in which the domains were presented was randomized. Participants could ask only three questions for each game: Across three rounds, they could select and click from among a set of six options displayed as buttons on the screen. The six options always included two constraint-seeking questions (e.g., “Does it live in the water?”)—one with higher and one with lower information gain, when possible—two pseudoconstraint-seeking questions (e.g., “Is it a flat kind of fish?”), and two hypothesis-scanning questions (e.g., “Is it the sole?”). Upon selection of a question, the computer automatically darkened the buttons corresponding to the objects the question had ruled out.

After participants had asked the third question, they had to guess the object the computer had randomly chosen by selecting one of the remaining objects. Children who guessed the right object in both games received a box of colored pencils. Adults who guessed both objects were entered in a lottery the winner of which was awarded a 20-Euro Amazon gift card.

### Results

We ran a repeated-measures ANOVA with the order of the questions (i.e., whether a question was asked in the first, second, or third round) and domain as within-subject factors, and inclusiveness and age group as between-subjects variables for all dependent measures. As in Study 2, when presenting the results for the type of questions selected, we report only the analysis of the proportion of constraint-seeking questions, for the sake of brevity. All main effects and interactions were tested but we report only significant effects.

#### Information gain

We found no effect of the order of questions (*p* = 0.13) or inclusiveness (*p* = 0.55) but a main effect of age group, *F*_(2, 82)_ = 18.6, *p* < 0.001, η^2^ = 0.31. A Bonferroni *post-hoc* analysis showed that adults selected questions with higher information gain (*M*_adults_ = 0.9; *SD* = 0.1) than older children (*M*_older_children_ = 0.7; *SD* = 0.2, *p* = 0.003), who selected questions with higher information gain than younger children (*M*_younger_children_ = 0.6; *SD* = 0.2, *p* = 0.021).

The analysis also showed a main effect of domain, *F*_(1, 82)_ = 4.5, *p* = 0.037, η^2^ = 0.05. Participants selected questions with higher information gain in the animals domain (*M*_animals_ = 0.79, *SD* = 0.02) than in the professions domain (*M*_professions_ = 0.75, *SD* = 0.02).

#### Question type

With the proportion of constraint-seeking questions as dependent variable, the analysis showed no effect of domain (*p* = 0.08), order of questions (*p* = 0.54), or inclusiveness (*p* = 0.58), but a main effect of age group, *F*_(2, 82)_ = 19.7, *p* < 0.001, η^2^ = 0.32. Bonferroni *post-hoc* analyses showed that adults selected a higher proportion of constraint-seeking questions (94%; *SD* = 14) than older children (69%; *SD* = 30; *p* = 0.002), who in turn selected a higher proportion of constraint-seeking questions than younger children (48%; *SD* = 36; *p* = 0.007).

### Discussion of study 3

The effect of object inclusiveness did not emerge in Study 3. In other words, domain and inclusiveness do not help or hinder participants in identifying and *selecting* more effective questions from a given set. This suggests that the facilitating effect of basic-level objects on categorization performance and the hindering effect of subordinate-level objects (Study 1) may have emerged only because of the types of features they trigger (Study 2). However, Study 3 also showed that to some extent all age groups were able to select the more effective constraint-seeking questions from a set of differentially effective questions. Whereas a random pick would have resulted in constraint-seeking questions in only about one third of the cases, even younger children picked the more effective question about 50% of the time.

## General discussion

In this paper we replicated and extended the investigation of the facilitating effect of basic-level objects on the ability to ask effective questions in a sequential binary categorization task (Study 1). Moreover, we aimed at providing a tentative explanation for this effect across different developmental stages, by disentangling the effect of the level of inclusiveness on the ability to *generate* features useful for categorization (Study 2), and on the ability to *select* the most efficient among a given set of questions addressing different categorization features (Study 3). To do so, we compared the effect of two different levels of object inclusiveness (basic-level and subordinate) in two object domains (animals and professions) on both formal quantitative and content-based qualitative measures of categorization performance of 7- and 10-year-olds, as well as adults.

Our experiments showed that the level of inclusiveness impacts the kinds of object-differentiating features participants think about (object-general vs. object-specific features) when reasoning about the objects. To differentiate animals/professions defined at the subordinate level from other animals/professions, participants in Study 1 and 2 could have referred to the object-general features that pertained to these objects and were generated by participants in the basic-level conditions. However, participants rarely considered object-general features when presented with subordinate-level objects. The fact that object-general features are more readily available in the basic-level condition made it more likely that participants ask effective categorization questions that help rule out more than one object at a time. In Study 3, we showed that the level of inclusiveness does not impact participants' ability to identify the most efficient from a set of given questions. This suggests that the level of inclusiveness at which objects are presented impact categorization performance mainly because of the kinds of features they trigger.

### Developmental implications

Our studies allow us to suggest a developmental trajectory of sequential binary categorization abilities. The performance pattern of our studies both confirmed and extended previous findings (e.g., Van Horn and Bartz, 1968; Denney and Denney, 1973; Siegler, 1977; Herwig, 1982; Ruggeri and Lombrozo, submitted). First, although all age groups seemed to benefit from basic-level objects (Studies 1 and 2), younger children were clearly limited by a lack of knowledge of a sufficient number of object-differentiating features (e.g., they “invented” contrasting features in Study 2). As in previous studies, Study 1 also showed that younger children needed more questions to reach the solution than older children and adults, and they asked fewer constraint-seeking questions with lower information gain than older children, who in turn asked fewer constraint-seeking questions than adults.

Interestingly, categorization performance improved across all age groups when participants were asked to choose from a set of differentially effective questions (Study 3). Thus, despite clear developmental differences, 7- and 10-year-olds seem to know what an effective categorization question looks like. Whereas younger children are mainly limited by their ability to generate enough object-differentiating features, older children are similar to adults in their ability to generate object-differentiating features but are still limited in their ability to generate and select those that are most effective for categorization (Studies 1 and 3). The developmental trajectory of these abilities should be taken in account when designing experiments where children have to generate questions or select among given questions.

In sum, the basic level of inclusiveness can help people of all ages to come up more readily with good categorization questions. However, with experience (e.g., as for adults) categorization performance remains robust independently of the level of inclusiveness and domain of the objects to be categorized.

### Practical implications

Our findings suggest that “cognitive algorithms… cannot be divorced from the information on which they operate and how that information is presented” (Gigerenzer and Hoffrage, 1995). Categorization is one of the most fundamental reasoning skills, deeply involved in decision-making, causal learning and probabilistic inferences. Our results can be made useful for teaching binary categorization (e.g., Martignon and Krauss, 2009) and to train categorization skills (e.g., Petursdottir et al., 2008). We suggest, based on our studies, that categorization problems should be set up using basic-level objects, because they tend to result in the most robust performance patterns across domains and age groups. Considering the strong domain differences, it is also crucial to start teaching categorization skills in commonly known domains. More generally, our studies point to an effective way to teach categorization based on the level of inclusiveness, that is, based on teaching people how to translate specific-level into basic-level descriptions and features rather than on teaching effective categorization strategies alone. In light of our results concerning how categorization can be improved without instruction, tutoring systems that enhance the idea of basic level of inclusiveness with instruction, explanation of strategies, and visual aids may prove most effective.

### Conflict of interest statement

The authors declare that the research was conducted in the absence of any commercial or financial relationships that could be construed as a potential conflict of interest.

## References

[B1] Atran S. (1990). Cognitive Foundations of Natural History: Towards an Anthropology of Science. New York, NY: Cambridge University Press.

[B2] Bauer P. J. Dow G. A. Hertzgaard L. A. (1995). Effects of prototypicality on categorization in 1- to 2-year-olds: getting down to basic. Cogn. Dev. 10, 43–68. 10.1016/0885-2014(95)90018-7

[B3] Berlin B. Breedlove D. E. Raven P. H. (1973). General principles of classification and nomenclature in folk biology. Am. Anthropol. 75, 214–242. 10.1525/aa.1973.75.1.02a00140

[B4] Berlin B. Breedlove D. Raven P. (1974). Principles of Tzeltal Plant Classification. New York, NY: Academic Press.

[B5] Berretty P. M. Todd P. M. Martignon L. (1999). Categorization by elimination: using few cues to choose, in Simple Heuristics that Make us Smart, eds Gigerenzer G. Todd P. M. The ABC Research Group (New York, NY: Oxford University Press), 235–254.

[B6] Bornstein M. H. (1984). A descriptive taxonomy of psychological categories used by infants, in Origins of Cognitive Skills, ed Sophian C. (Hillsdale: Erlbaum), 313–338.

[B7] Bornstein M. H. Arterberry M. E. (2010). The development of object categorization in young children: hierarchical inclusiveness, age, perceptual attribute, and group versus individual analyses. Dev. Psychol. 46, 350–365. 10.1037/a0018411 20210495 PMC2856652

[B8] Bornstein M. H. Kessen W. Weiskopf S. (1976). Color vision and hue categorization in young human infants. J. Exp. Psychol. Hum. 2, 115–129. 10.1037/0096-1523.2.1.115 1262792

[B9] Bulmer R. (1974). Folk biology in the New Guinea Highlands. Soc. Sci. Inform. 13, 9–28. 10.1177/053901847401300402

[B10] Boy Scouts of America. (1911). Boy Scouts Handbook. New York, NY: New York Dover.

[B11] Chouinard M. M. (2007). Children's questions: a mechanism for cognitive development. Monogr. Soc. Res. Child Dev. 72, vii–ix. 10.1111/j.1540-5834.2007.00412.x 17394580

[B12] Denney D. R. Denney N. W. (1973). The use of classification for problem solving: a comparison of middle and old age. Dev. Psychol. 9, 275–278. 10.1037/h0035092

[B13] Dougherty J. W. D. (1978). Salience and relativity in classification. Am. Ethnol. 5, 66–68. 10.1525/ae.1978.5.1.02a00060

[B14] Ellen R. (1993). The Cultural Relations of Classification. Cambridge: Cambridge University Press.

[B15] Ellis A. E. Oakes L. M. (2006). Infants flexibly use different dimensions to categorize objects. Dev. Psychol. 42, 1000–1011. 10.1037/0012-1649.42.6.1000 17087536

[B16] Flavell J. (1985). Cognitive Development, 2nd Edn Englewood Cliffs: Prentice-Hall.

[B17] Gigerenzer G. Hoffrage U. (1995). How to improve Bayesian reasoning without instruction: frequency formats. Psychol. Rev. 102, 684–704. 10.1037/0033-295X.102.4.68426030173

[B18] Green L. Mehr D. R. (1997). What alters physicians' decisions to admit to the coronary care unit? J. Fam. Pract. 45, 219–226. 9300001

[B19] Hamilton G. C. Sanders A. Strange G. S. Trott A. T. (2003). Emergency Medicine: An Approach to Clinical Problem Solving, 2nd Edn Philadelphia, PA: Saunders Co.

[B20] Herwig J. E. (1982). Effects of age, stimuli, and category recognition factors in children's inquiry behavior. J. Exp. Child Psychol. 33, 196–206. 10.1016/0022-0965(82)90015-7 7069364

[B21] Hund A. M. Plumert J. M. (2005). The stability and flexibility of spatial categories. Cogn. Psychol. 50, 1–44. 10.1016/j.cogpsych.2004.05.002 15556128

[B22] Hunn E. (1982). The utilitarian factor in folk biological classification. Am. Anthropol. 84, 830–847. 10.1525/aa.1982.84.4.02a00070

[B23] John D. R. Sujan M. (1990). Children's use of perceptual cues in product categorization. Psychol. Market. 7, 277–294. 10.1002/mar.4220070404

[B24] Johnson K. E. Mervis C. B. (1997). Effects of varying levels of expertise on the basic level of categorization. J. Exp. Psychol. Gen. 126, 248–277. 10.1037/0096-3445.126.3.248 9281832

[B25] Legare C. H. Mills C. M. Souza A. L. Plummer L. E. Yasskin R. (2013). The use of questions as problem-solving strategies during early childhood. J. Exp. Child Psychol. 114, 63–76. 10.1016/j.jecp.2012.07.002 23044374

[B26] Lindley D. V. (1956). On a measure of the information provided by an experiment. Ann. Math. Stat. 27, 986–1005. 10.1214/aoms/1177728069

[B27] Liu J. Golinkoff R. M. Sak K. (2001). One cow does not an animal make: young children can extend novel words at the superordinate level. Child Dev. 72, 1674–1694. 10.1111/1467-8624.00372 11768139

[B28] Mandler J. M. Bauer P. J. (1988). The cradle of categorization: is the basic level basic? Cogn. Dev. 3, 247–264. 10.1016/0885-2014(88)90011-1

[B29] Mareschal D. Tan S. H. (2007). Flexible and context-dependent categorization by eighteen-month-olds. Child Dev. 78, 19–37. 10.1111/j.1467-8624.2007.00983.x 17328691

[B30] Martignon L. Katsikopoulos K. V. Woike J. K. (2008). Categorization with limited resources: a family of simple heuristics. J. Math. Psychol. 52, 352–361. 10.1016/j.jmp.2008.04.003

[B31] Martignon L. Krauss S. (2009). Hands-on activities for fourth graders: a tool box for decision-making and reckoning with risk. Int. Electron. J. Math. Educ. 4, 227–258.

[B32] Medin D. L. Lynch E. B. Coley J. D. Atran S. (1997). Categorization and reasoning among tree experts: do all roads lead to rome? Cogn. Psychol. 32, 49–96. 10.1006/cogp.1997.0645 9038245

[B33] Mervis C. B. (1987). Child-basic object categories and early lexical development, in Concepts and Conceptual Development: Ecological and Intellectual Factors in Categorization, ed Neisser U. (New York, NY: Cambridge University Press), 201–233.

[B34] Mervis C. B. Crisafi C. A. (1982). Order of acquisition of subordinate-, basic-, and superordinate-level categories. Child Dev. 53, 267–273. 10.2307/1129661

[B35] Mervis C. B. Rosch E. (1981). Categorization of natural objects. Annu. Rev. Psychol. 32, 89–115. 10.1146/annurev.ps.32.020181.000513

[B36] Mosher F. A. Hornsby J. R. (1966). On asking questions, in Studies in Cognitive Growth, eds Bruner J. S. Oliver R. R. Greenfield P. M. (New York, NY: Wiley), 86–101.

[B37] Murphy G. L. (2002). The Big Book of Concepts. Cambridg: MIT Press.

[B38] Nelson J. D. (2005). Finding useful questions: on Bayesian diagnosticity, probability, impact and information gain. Psychol. Rev. 112, 979–999. 10.1037/0033-295X.112.4.979 16262476

[B39] Nelson J. D. Divjak B. Martignon L. Gudmundsdottir G. Meder B. (2014). Children's sequential information search is sensitive to environmental probabilities. Cognition 130, 74–80. 10.1016/j.cognition.2013.09.007 24184396

[B40] Oakes L. M. Coppage D. J. Dingel A. (1997). By land or by sea: the role of perceptual similarity in infants' categorization of animals. Dev. Psychol. 33, 396–407. 10.1037/0012-1649.33.3.396 9149919

[B41] Oakes L. M. Plumert J. M. Lansink J. M. Merryman J. D. (1996). Evidence for task-dependent categorization in infancy. Infant Behav. Dev. 19, 425–444. 10.1016/S0163-6383(96)90004-1

[B42] Oakes L. M. Ribar R. J. (2005). A comparison of infants' categorization in paired and successive presentation familiarization tasks. Infancy 7, 85–98. 10.1207/s15327078in0701_7 33430540

[B43] Oaksford M. Chater N. (1994). A rational analysis of the selection task as optimal data selection. Psychol. Rev. 101, 608–631. 10.1037/0033-295X.101.4.608

[B44] Oaksford M. Chater N. (1996). Rational explanation of the selection task. Psychol. Rev. 103, 381–391. 10.1037/0033-295X.103.2.381

[B45] Palmer C. F. Jones R. K. Hennessy B. L. Unze M. G. Pick A. D. (1989). How is a trumpet known? The “basic object level” concept and the perception of musical instruments. Am. J. Psychol. 102, 17–37. 2929787

[B46] Petursdottir A. I. Carr J. E. Lechago S. A. Almason S. M. (2008). An evaluation of intraverbal training and listener training for teaching categorization skills. J. Appl. Behav. Anal. 41, 53–68. 10.1901/jaba.2008.41-53 18468279 PMC2410193

[B47] Rakison D. H. Oakes L. (2003). Early Category and Concept Development. New York, NY: Oxford University Press.

[B48] Rosch E. (1978). Principles of categorization, in Cognition and Categorization, eds Rosch E. Lloyd B. B. (Hillsdale: Erlbaum), 27–48.

[B49] Rosch E. Mervis C. B. Gray W. Johnson D. Boyes-Braem P. (1976). Basic objects in natural categories. Cogn. Psychol. 8, 382–439. 10.1016/0010-0285(76)90013-X

[B50] Ruggeri A. Lombrozo T. (2014). Learning by asking: how children ask questions to achieve efficient search, in Proceedings of the 36th Annual Conference of the Cognitive Science Society, eds Bello P. Guarini M. McShane M. Scassellati B. (Austin, TX: Cognitive Science Society), 1335–1340.

[B51] Ruggeri A. Lombrozo T. Griffiths T. L. Xu F. (2015). Children search for information as efficiently as adults, but seek additional confirmatory evidence, in Proceedings of the 37th Annual Conference of the Cognitive Science Society.

[B52] Schyns P. G. Rodet L. (1997). Categorization creates functional features. J. Exp. Psychol. Learn. 23, 681–696. 10.1037/0278-7393.23.3.681

[B53] Shannon C. E. (1948). A mathematical theory of communication. Bell Syst. Tech. J. 27, 379–423, 623–656.

[B54] Siegler R. S. (1977). The twenty questions game as a form of problem solving. Child Dev. 48, 395–403. 10.2307/1128632

[B55] Springer K. (2001). Perceptual boundedness and perceptual support in conceptual development. Psychol. Rev. 108, 691–708. 10.1037/0033-295X.108.4.691 11699113

[B56] Tanaka J. W. Taylor M. E. (1991). Categorization and expertise: is the basic level in the eye of the beholder? Cogn. Psychol. 23, 457–482. 10.1016/0010-0285(91)90016-H

[B57] Van Horn K. R. Bartz W. H. (1968). Information seeking strategies in cognitive development. Psychon. Sci. 11, 341–342. 10.3758/BF03328225

[B58] Wartella E. (1979). Children Communicating: Media and Development of Thought, Speech, Understanding. Beverly Hills: Sage.

[B59] Wisniewski E. J. Murphy G. L. (1989). Superordinate and basic category names in discourse: a textual analysis. Discourse Process. 12, 245–261. 10.1080/01638538909544728

[B60] Younger B. A. Furrer S. (2003). A comparison of visual familiarization and object-examining measures of categorization in 9-month-old infants. Infancy 4, 327–348. 10.1207/S15327078IN0403_02

