# Supplementary material for: How basic-level objects facilitate question-asking in a categorization task
Source: Front Psychol. 2015 Jul 10;6:918. doi: 10.3389/fpsyg.2015.00918 (PMC4498020; doi:10.3389/fpsyg.2015.00918)
Supplement: Supplementary file 1 [file Table1.DOC]

Appendix 1. *Objects Used in Studies 1, 2 and 3, in Italian with English Translation, Listed by Domain (Animal, Profession) and Condition (Basic Level, Subordinate).*

| Animals domain | | Professions domain | |
| --- | --- | --- | --- |
| Basic level | Subordinate level | Basic level | Subordinate level |
| Pecora - *Sheep* | Agnello *Lamb* | Dottore- *Doctor* | Dentista - *Dentist* |
| Cane *- Dog* | Dalmata - *Dalmatian* | Insegnante - *Teacher* | Maestro elementare - *Kindergarten teacher* |
| Gatto - *Cat* | Persiano - *Persian* | Cuoco - *Cook* | Pasticciere - *Confectioner* |
| Mucca - *Cow* | Vitello - *Calf* | Poliziotto - *Police officer* | Investigatore - *Detective* |
| Cavallo -*Horse* | Pony - *Pony* | Cantante - *Singer* | Soprano - *Soprano* |
| Mosca - *Fly* | Moscerino - *Gnat* | Allevatore - *Breeder* | Pastore - *Shepherd* |
| Uccello - *Bird* | Piccione - *Pigeon* | Atleta - *Athlete* | Ciclista - *Cyclist* |
| Dinosauro -*Dinosaur* | Tirannosauro - *Tyrannosaurus* | Fotografo -*Photographer* | Fotogiornalista - *Photojournalist* |
| Serpente - *Snake* | Vipera - *Viper* | Musicista - *Musician* | Flautista - *Flautist* |
| Orso - *Bear* | Panda - *Panda* | Attore - *Actor* | Teatrante - *Theater actor* |
| Scimmia - *Ape* | Gorilla - *Gorilla* | Soldato - *Soldier* | Paracadutista - *Parachutist* |
| Ape - *Bee* | Ape regina - *Queen bee* | Agricoltore - *Farmer* | Viticoltore -*Winegrower* |
| Ragno - *Spider* | Vedova nera - *Black widow* | Artigiano - *Craftsman* | Falegname - *Carpenter* |
| Farfalla - *Butterfly* | Falena - *Moth* | Autista - *Driver* | Autista di autobus - *Bus driver* |
| Pollo - *Chicken* | Gallina - *Chick* | Circense - *Circus performer* | Giocoliere - *Juggler* |
| Maiale - *Pig* | Cinghiale - *Boar* | Politico - *Politician* | Sindaco - *Mayor* |
| Rana - *Frog* | Girino - *Tadpole* | Disegnatore - *Drawer* | Fumettista - *Cartoonist* |
| Granchio - *Crab* | Favollo - *Warty crab* | Giornalista - *Journalist* | Reporter - *Sports reporter* |
| Pesce - *Fish* | Sogliola - *Sole* | Allenatore - *Coach* | Mister - *Soccer coach* |
| Coniglio - *Rabbit* | Lepre - *Hare* | Commesso - *Salesperson* | Fioraio - *Florist* |
