# Supplementary material for: How basic-level objects facilitate question-asking in a categorization task
Source: Front Psychol. 2015 Jul 10;6:918. doi: 10.3389/fpsyg.2015.00918 (PMC4498020; doi:10.3389/fpsyg.2015.00918)
Supplement: Supplementary file 2 [file Table2.DOCX]

Appendix 2*. Italian Instructions of Study 1*

“Dimmi tutto ciò che rende il cane [Dalmata/dottore/dentista] diverso da questi altri animali [mestieri], tutto ciò che lo distingue.”
